# Concurrent pretreatment serum and BALF galactomannan positivity as a prognostic indicator in non-neutropenic invasive pulmonary aspergillosis without malignancy or solid organ transplantation: a retrospective cohort study

**DOI:** 10.3389/fmed.2026.1809671

**Published:** 2026-05-13

**Authors:** DeZhu Tang, Qian Xiong, YangLingXi Wang, XiaoYing Luo, JinWei Jia, YanMei Feng, WeiDuo Zhou

**Affiliations:** 1 Department of Respiratory Medicine, Chongqing University Central Hospital, Chongqing, China; 2 Department of Neurosurgery, Chongqing University Central Hospital, Chongqing, China; 3 Department of Respiratory and Critical Care Medicine, The First Affiliated Hospital of Chongqing Medical University, Chongqing, China

**Keywords:** galactomannan, invasive pulmonary aspergillosis, mortality, non-neutropenic, prognosis, retrospective cohort study

## Abstract

**Background:**

The prognostic evaluation of invasive pulmonary aspergillosis (IPA) in non-neutropenic patients remains clinically challenging. While galactomannan (GM) testing in serum and bronchoalveolar lavage fluid (BALF) aids in diagnosis, the prognostic significance of concurrent positivity in both compartments is not well-established. This study aimed to determine whether concurrent pretreatment GM positivity serves as an independent predictor of 30-day all-cause mortality in non-neutropenic IPA patients without underlying malignancy or solid organ transplantation.

**Methods:**

We conducted a single-center, retrospective cohort study including 162 patients with proven or probable IPA admitted between February 2018 and February 2025. Patients were stratified into two groups based on stringent, predefined criteria: the GM-positive group (concurrent pretreatment serum GM index ≥ 0.7 and BALF GM index ≥ 0.8, *n* = 81) and the GM-negative group (both indices below these diagnostic thresholds, *n* = 81). All patients received standardized first-line antifungal therapy (voriconazole). The primary outcome was 30-day all-cause mortality. Survival analysis was performed using Kaplan-Meier estimates and Cox proportional hazards models, with adjustment for relevant clinical confounders.

**Results:**

The overall 30-day mortality was 29.6% (48/162). Mortality was significantly higher in the GM-positive group compared to the GM-negative group (45.7% vs. 13.6%, *P* < 0.001). Multivariable Cox regression analysis confirmed that concurrent pretreatment GM positivity was independently associated with increased mortality (adjusted hazard ratio [HR] = 3.07; 95% confidence interval [CI], 1.5–6.29; *P* = 0.002), after adjusting for factors including APACHE II score and key laboratory parameters.

**Conclusion:**

In non-neutropenic IPA patients without classic immunocompromising conditions (e.g., malignancy or solid organ transplantation), concurrent pretreatment positivity for both serum and BALF GM is an independent prognostic marker for increased 30-day all-cause mortality. This combined assessment may serve as a valuable tool for early risk stratification, potentially helping to identify a high-risk subgroup that could benefit from more vigilant monitoring and individualized management strategies.

## 1 Introduction

Invasive aspergillosis (IA) is a life-threatening fungal infection with an estimated global incidence of over 300,000 cases per year and mortality rates ranging from 30% to 80% (1). Its most common manifestation is invasive pulmonary aspergillosis (IPA). While traditionally associated with profound neutropenia, IPA is increasingly recognized in non-neutropenic patients with underlying conditions such as chronic obstructive pulmonary disease (COPD), diabetes mellitus (DM), liver cirrhosis, or following severe viral respiratory infections (e.g., influenza, COVID-19) (2–6). In these populations, diagnosis is often delayed due to atypical presentations, contributing to poor outcomes (5, 6). Consequently, early identification and accurate risk stratification are paramount for improving IPA management.

Galactomannan (GM), a polysaccharide component of the *Aspergillus* cell wall, is a key biomarker incorporated into the diagnostic criteria for invasive fungal disease established by the European Organization for Research and Treatment of Cancer and the Mycoses Study Group Education and Research Consortium (EORTC/MSGERC) (7). GM can be detected in both serum and bronchoalveolar lavage fluid (BALF) (8). BALF GM generally exhibits higher and more consistent sensitivity for IPA across diverse patient groups compared to serum GM, the latter being more variable and dependent on the degree of angioinvasion and host immune status (9–13).

Despite its established diagnostic role, the prognostic value of GM testing, particularly in non-neutropenic patients without underlying malignancy or solid organ transplantation, remains controversial and inadequately studied. Most prior investigations have evaluated serum or BALF GM in isolation, yielding inconsistent results regarding their association with mortality (13–18). We hypothesized that concurrent positivity in both compartments might delineate a distinct, high-risk disease phenotype. BALF GM positivity likely reflects a substantial local fungal burden in the lungs, whereas serum GM positivity may indicate early systemic dissemination. The combination of both could therefore signal a more aggressive infection.

To test this hypothesis, we conducted a retrospective cohort study focusing on a well-defined population of non-neutropenic IPA patients without underlying malignancy or solid organ transplantation. We aimed to determine whether concurrent pretreatment positivity for serum and BALF GM is independently associated with 30-day all-cause mortality, with the goal of providing evidence for a simple, biomarker-based strategy for early prognostic stratification.

## 2 Materials and methods

### 2.1 Study population

Clinical data were retrospectively collected from patients diagnosed with IPA in Chongqing University Central Hospital, between February 2018 and February 2025. The dataset included demographic information and admission laboratory biomarkers. The study protocol was reviewed and approved by the Ethics Committee of Chongqing University Central Hospital.

Patients were eligible for inclusion if they were aged 18 years or older, non-pregnant, and diagnosed with IPA according to the 2020 EORTC/MSGERC (7) criteria and the BM-AspICU algorithm (19). The diagnostic framework incorporated the following elements: host factors (e.g., prolonged corticosteroid use (≥0.3 mg/kg for ≥3 weeks within the preceding 60 days), COPD, viral respiratory infections such as influenza or SARS-CoV-2, liver cirrhosis, DM, or a history of cardiac surgery), compatible clinical manifestations (e.g., persistent fever despite ≥ 3 days of antibiotic therapy, cough, sputum production, pleuritic chest pain, dyspnea, hemoptysis, or respiratory failure even with ventilatory support), radiological features on chest computed tomography (e.g., air crescent sign, cavitary lesions with or without a halo sign, well-defined nodules, diffuse reticulonodular or alveolar infiltrates, wedge-shaped or lobar consolidation, pleural effusion, or tree-in-bud appearance), and mycological/histopathological evidence.Critically, mycological evidence for proven or probable IPA was fulfilled by any of the following: microscopic or culture positivity for *Aspergillus* species in qualified respiratory specimens, positive polymerase chain reaction (PCR) results from blood and/or BALF, or lung biopsy demonstrating hyphae with tissue invasion. This allowed for diagnosis in GM-negative patients via alternative mycological methods (e.g., culture or PCR). Based on these criteria, cases were classified as proven IPA (if histopathological evidence was available) or probable IPA (if host, clinical, radiological, and mycological criteria were satisfied). Additional inclusion requirements consisted of receipt of voriconazole as standardized first-line antifungal therapy according to institutional protocol, initiated immediately after diagnosis; availability of both serum and BALF GM testing within 72 hs prior to antifungal initiation; and complete clinical follow-up including survival data.

Patients were excluded for any of the following reasons: a single serum or BALF GM optical density index (ODI) ≥ 1.0; coinfection with other invasive fungal diseases (e.g., mucormycosis or cryptococcosis); death before initiating antifungal therapy; or receipt of antifungal agents prior to IPA diagnosis. We also excluded patients with neutropenia (absolute neutrophil count < 0.5 × 10^9^/L), underlying hematologic or solid malignancies, or a history of solid organ transplantation. Additionally, exclusion criteria encompassed cases with potential sources of GM false positivity (such as recent β-lactam antibiotic use or albumin infusion), *Aspergillus* colonization, chronic or allergic bronchopulmonary aspergillosis, or incomplete clinical data.

All eligible patients meeting the predefined inclusion and exclusion criteria during the study period were screened. Patients with concurrent pretreatment positivity for both serum and BALF GM were all included. During the same period, the number of patients with concurrent negativity in both serum and BALF GM who met the same eligibility criteria was identical. No matching, stratification, or selective sampling was applied. Therefore, the equal sample size between the two groups arose naturally from the underlying cohort rather than from any deliberate balancing strategy.

### 2.2 Clinical data

Demographic and clinical characteristics were retrospectively collected for all enrolled patients with IPA. Baseline characteristics included age, sex, and comorbidities such as hypertension, DM, COPD, and concurrent bacterial pneumonia. Additional data regarding smoking history, requirements for mechanical ventilation, oxygenation index (OI), and Acute Physiology and Chronic Health Evaluation II (APACHE II) score were also recorded.

At admission, laboratory measurements included albumin (ALB), serum creatinine (SCr), glucose (Glu), lactate dehydrogenase (LDH), white blood cell count (WBC), neutrophil count, lymphocyte count, hemoglobin (HGB), platelet count (PLT), eosinophil count, interleukin-6 (IL-6), procalcitonin (PCT), and C-reactive protein (CRP). Microbiological findings, including sputum culture, BALF culture and indirect immunofluorescence assay (IFA) results, were also collected from the medical records.

### 2.3 Sample collection, GM testing, and group definition

Bronchoalveolar lavage fluid samples were obtained via flexible bronchoscopy performed by experienced pulmonologists (all with ≥5 years of practice). Lavage was targeted at bronchopulmonary segments corresponding to radiological abnormalities on chest imaging. All serum and BALF samples were collected prior to the initiation of any antifungal therapy. GM testing was performed on both sample types in strict accordance with the manufacturer’s protocols (KREDI Biotech, Jiaxing, China).

The GM ODI was calculated as the sample absorbance value divided by the assay’s cutoff optical density. Patients were stratified based on their pretreatment GM status. In alignment with the 2020 EORTC/MSGERC criteria (7), concurrent positivity was defined as a serum GM ODI ≥ 0.7 and a BALF GM ODI ≥ 0.8. Patients meeting both thresholds were designated as the GM-positive group, while those with both serum and BALF GM ODI below these cut-offs constituted the GM-negative group. To specifically evaluate the prognostic effect of dual-compartment positivity and minimize heterogeneity, patients with an isolated positive GM result (i.e., only serum or only BALF GM ODI ≥ 1.0) were excluded from the analysis.

### 2.4 Statistical analysis

Descriptive analyses were performed for all included patients. Categorical variables were summarized as counts and percentages, whereas continuous variables were reported as mean (SD) if normally distributed or as median (interquartile range, IQR) if skewed.

The association between concurrent pretreatment GM status and 30-day mortality was assessed using univariate and multivariable Cox proportional hazards regression models. Results are presented as hazard ratios (HRs) with corresponding 95% confidence intervals (CIs). A 2-sided *P*-value < 0.05 was considered statistically significant.

Survival differences between groups stratified by GM status were further examined using Kaplan-Meier survival curves, with statistical significance evaluated by the log-rank test.

Subgroup analyses were conducted to explore potential effect modification across prespecified clinical variables, including age (<65 vs. ≥65 years), DM (yes vs. no), COPD (yes vs. no), hypertension (yes vs. no), smoking status (yes vs. no), and mechanical ventilation (yes vs. no). Stratified Cox regression models and likelihood ratio tests were used to assess interactions.

Patients with missing data for key variables (including serum GM, BALF GM, or survival outcomes) were excluded from the final analysis. All statistical analyses were performed using R software (R Foundation for Statistical Computing^1^) and Free Statistics software, version 1.9.

## 3 Results

### 3.1 Baseline characteristics

**FIGURE 1:**
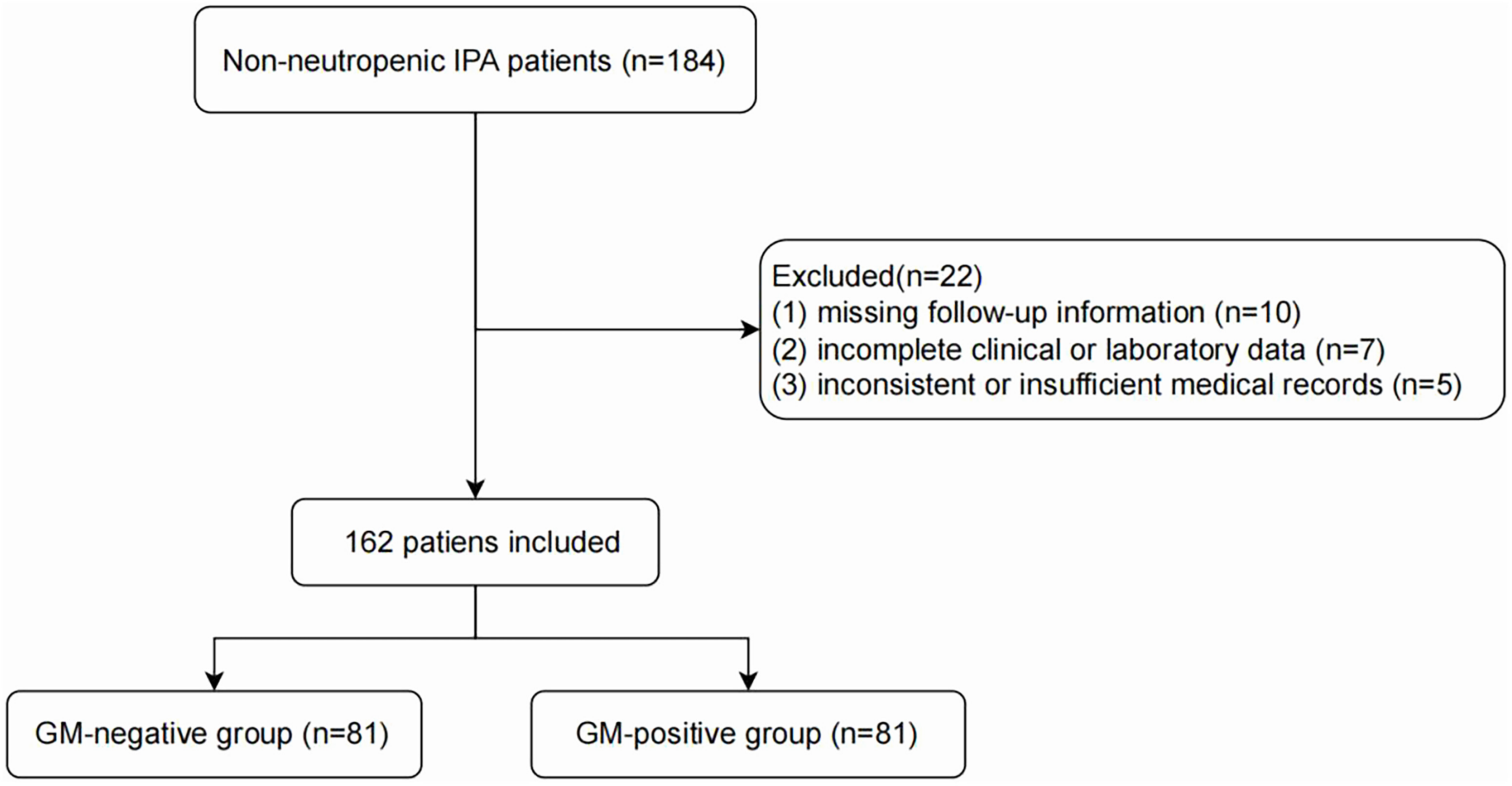
Flow chart of the study population.

From the 184 patients who initially met the inclusion criteria, 22 were excluded, resulting in a final analytic cohort of 162 patients with IPA. According to the EORTC/MSGERC criteria and the BM-AspICU algorithm (7, 19), six patients (3.7%) were classified as having proven IPA and 156 (96.3%) as probable IPA, reflecting the limited use of invasive tissue sampling in this patient population. Specifically, in the GM-positive group (*n* = 81), there were two cases (2.5%) of proven IPA and 79 cases (97.5%) of probable IPA; in the GM-negative group (*n* = 81), there were four cases (4.9%) of proven IPA and 77 cases (95.1%) of probable IPA. The cohort comprised 81 patients with concurrent pretreatment GM positivity and 81 patients with concurrent GM negativity; no matching procedures were performed (Figure 1). The baseline characteristics of the cohort are summarized in Table 1. The study population had a mean (SD) age of 66.4 (13.5) years, ranging from 20 to 94, and included 37 females (22.8%). The most common comorbidity was hypertension (77 [47.5%]), followed by DM (58 [35.8%]), COPD (53 [32.7%]), and bacterial pneumonia (50 [30.9%]). Ninety patients (55.6%) had a history of smoking, and 47 (29.0%) required mechanical ventilation during hospitalization. The mean OI was 252.8 ± 101.2 mmHg, and the mean APACHE II score was 14.5 ± 7.9.

**TABLE 1:** Baseline characteristics of participants.

Variables	Total (*n* = 162)	Concurrent pretreatment GM status		*P*-value
		Negative(*n* = 81)	Positive (*n* = 81)	
Demographic and clinical characteristics				
Age (years)	66.4 ± 13.5	66.5 ± 15.8	66.2 ± 10.9	0.858
Sex, female, *n* (%)	37 (22.8)	21 (25.9)	16 (19.8)	0.349
Hypertension, *n* (%)	77 (47.5)	36 (44.4)	41 (50.6)	0.431
DM, *n* (%)	58 (35.8)	27 (33.3)	31 (38.3)	0.512
COPD, *n* (%)	53 (32.7)	25 (30.9)	28 (34.6)	0.615
Bacterial pneumonia, *n* (%)	50 (30.9)	20 (24.7)	30 (37)	0.089
Smoking, *n* (%)	90 (55.6)	49 (60.5)	41 (50.6)	0.206
Mechanical ventilation, *n* (%)	47 (29.0)	23 (28.4)	24 (29.6)	0.863
OI (mmHg)	252.8 ± 101.2	261.9 ± 101.6	243.7 ± 100.6	0.253
APACHE II score, *n* (%)	14.5 ± 7.9	13.2 ± 7.2	15.8 ± 8.3	0.036
Laboratory values				
ALB (g/L)	33.7 ± 5.5	35.0 ± 5.3	32.4 ± 5.4	0.002
SCr (μmoI/L)	81.0 (61.0, 155.7)	74.0 (57.0, 107.0)	95.0 (63.0, 206.0)	0.002
Glu (mmol/L)	9.1 (6.6, 12.7)	8.2 (6.0, 11.5)	10.4 (7.5, 15.0)	0.002
LDH (U/L)	258.0 (198.2, 380.0)	243.0 (180.0, 356.0)	292.0 (230.0, 408.0)	0.052
WBC (× 10^9^/L)	9.3 (6.3, 14.7)	8.8 (6.1, 13.7)	9.8 (7.2, 16.1)	0.143
Neu# (× 10^9^/L)	8.1 (5.0, 13.3)	7.0 (4.6, 12.1)	8.7 (5.6, 14.5)	0.075
Lym# (× 10^9^/L)	0.7 (0.4, 1.3)	0.7 (0.4, 1.1)	0.7 (0.4, 1.3)	0.642
HGB (g/L)	114.3 ± 28.0	118.3 ± 27.5	110.3 ± 28.1	0.067
PLT (× 10^9^/L)	169.5 (123.2, 224.2)	163.0 (117.0, 236.0)	174.0 (131.0, 213.0)	0.568
EO (× 10^9^/L)	0.01 (0.01, 0.04)	0.01 (0.01, 0.04)	0.01 (0.01, 0.04)	0.568
IL-6 (pg/ml)	109.9 (32.1, 402.6)	66.2 (17.2, 157.3)	183.6 (66.2, 601.9)	0.036
PCT (ng/ml)	0.3 (0.1, 3.0)	0.1 (0.1, 0.8)	1.0 (0.2, 7.6)	0.368
CRP (mg/L)	65.6 (24.7, 118.9)	49.5 (18.0, 98.4)	87.4 (41.8, 134.4)	0.009
Microbiological findings				
IFA, *n* (%)	40 (24.7)	12 (14.8)	28 (34.6)	0.004
SC, *n* (%)	96 (59.3)	42 (51.9)	54 (66.7)	0.055
BALF culture, *n* (%)	94(58.0)	43(53.1)	51(63.0)	0.203
30-day mortality, *n* (%)	48 (29.6)	11 (13.6)	37 (45.7)	<0.001

GM, galactomannan; DM, diabetes mellitus; COPD, chronic obstructive pulmonary disease; OI, oxygenation index; APACHE II, Acute Physiology and Chronic Health Evaluation II; ALB, albumin; SCr, serum creatinine; Glu, glucose; LDH, lactate dehydrogenase; WBC, white blood cell count; Neu#,neutrophil count; Lym#, lymphocyte count; HGB, hemoglobin; PLT, platelet count; EO, eosinophil count; IL-6, interleukin-6; PCT, procalcitonin; CRP, C-reactive protein; IFA, immunofluorescence assay; SC, sputum culture; BALF, bronchoalveolar lavage fluid.

Overall, 48 patients (29.6%) died within 30 days. Mortality was substantially higher in the GM-positive group than in the GM-negative group (37 [45.7%] vs. 11 [13.6%]; *P* < 0.001). Patients in the GM-positive group had a significantly higher positivity rate of IFA, whereas no significant differences were observed in sputum and BALF culture positivity between the two groups. Additional baseline laboratory parameters are summarized in Table 1.

### 3.2 Association of concurrent pretreatment GM status with 30-day mortality

In univariable Cox regression analyses, several baseline factors were significantly associated with 30-day mortality, including hypertension, DM, mechanical ventilation, APACHE II score, albumin, serum creatinine, glucose, lactate dehydrogenase, white blood cell count, neutrophil count, hemoglobin, interleukin-6, C-reactive protein, IFA and concurrent pretreatment GM positivity (all *P* < 0.05; Supplementary Table 1).

Covariates with *P* < 0.05 in univariable analyses were considered for multivariable modeling. To avoid overfitting, collinearity and coefficient stability were evaluated by comparing crude and fully adjusted models, and only a limited number of clinically representative and statistically stable covariates were retained. The number of covariates was restricted according to the number of outcome events, and results from fully adjusted models are provided in the Supplementary Table 2.

When these variables were entered into a multivariable Cox regression model, concurrent pretreatment GM positivity remained independently associated with increased 30-day mortality (adjusted hazard ratio, 3.07; 95% CI, 1.5–6.29; *P* = 0.002) (Table 2).

**TABLE 2:** Multivariable cox regression analysis of concurrent pretreatment GM status and 30-day mortality.

*n*. total	Adjusted HR	95% CI	*P*-value
162	3.07	(1.5∼6.29)	0.002

Adjusted covariates: APACHE II score, ALB, SCr, Glu. GM, galactomannan; APACHE II, Acute Physiology and Chronic Health Evaluation II; ALB, albumin; SCr, serum creatinine; Glu, glucose.

**FIGURE 2:**
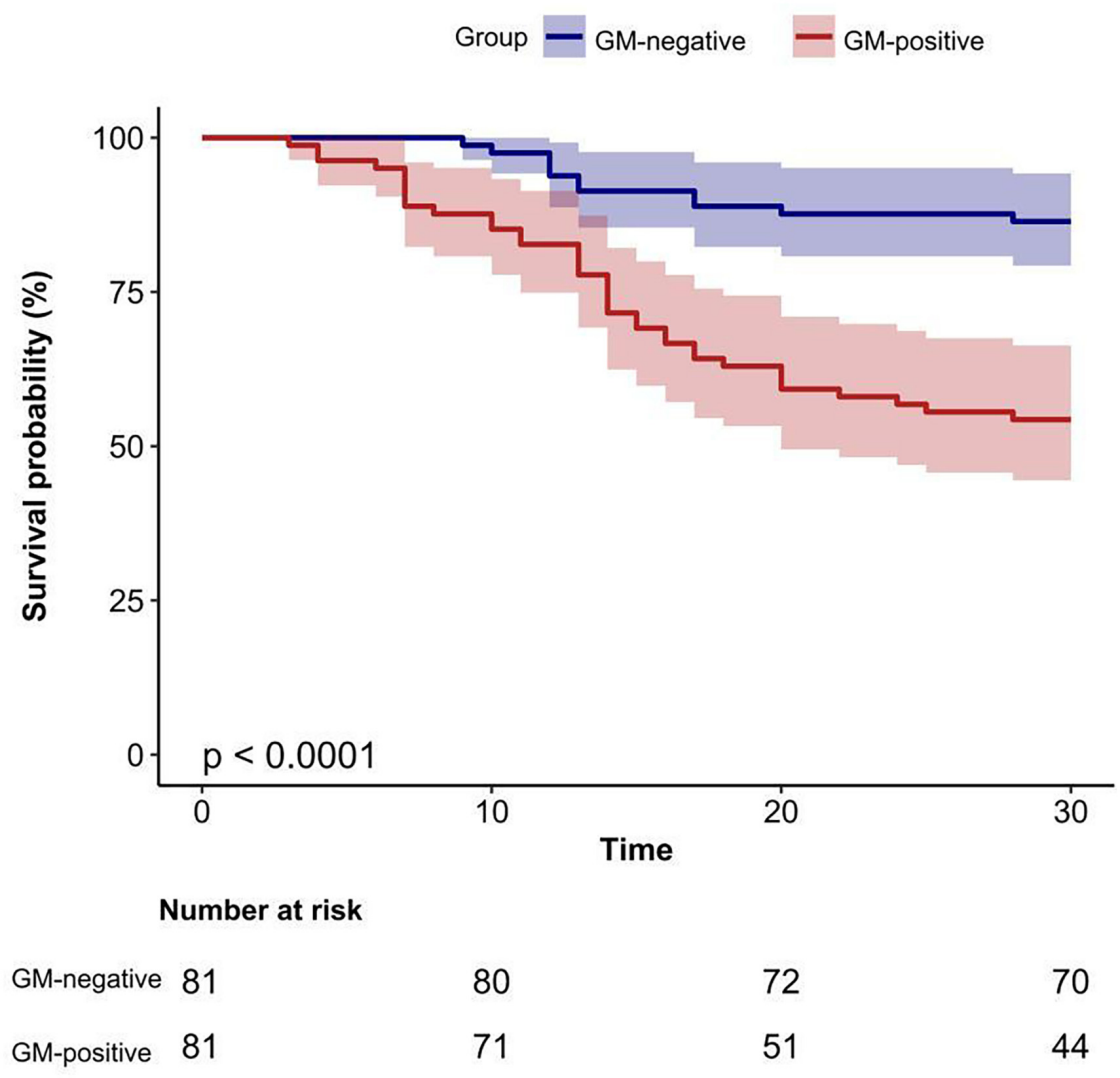
Kaplan-Meier curves for 30-day survival according to GM status. GM-positive: serum GM (ODI) ≥ 0.7 and BALF GM (ODI) ≥ 0.8; GM-negative: values below both thresholds.GM, galactomannan; ODI, optical density index; BALF, bronchoalveolar lavage fluid.

Kaplan-Meier survival analysis further demonstrated significantly higher 30-day mortality in the GM-positive group compared with the GM-negative group (log-rank test, *P* < 0.0001) (Figure 2).

### 3.3 Subgroup analyses

**FIGURE 3:**
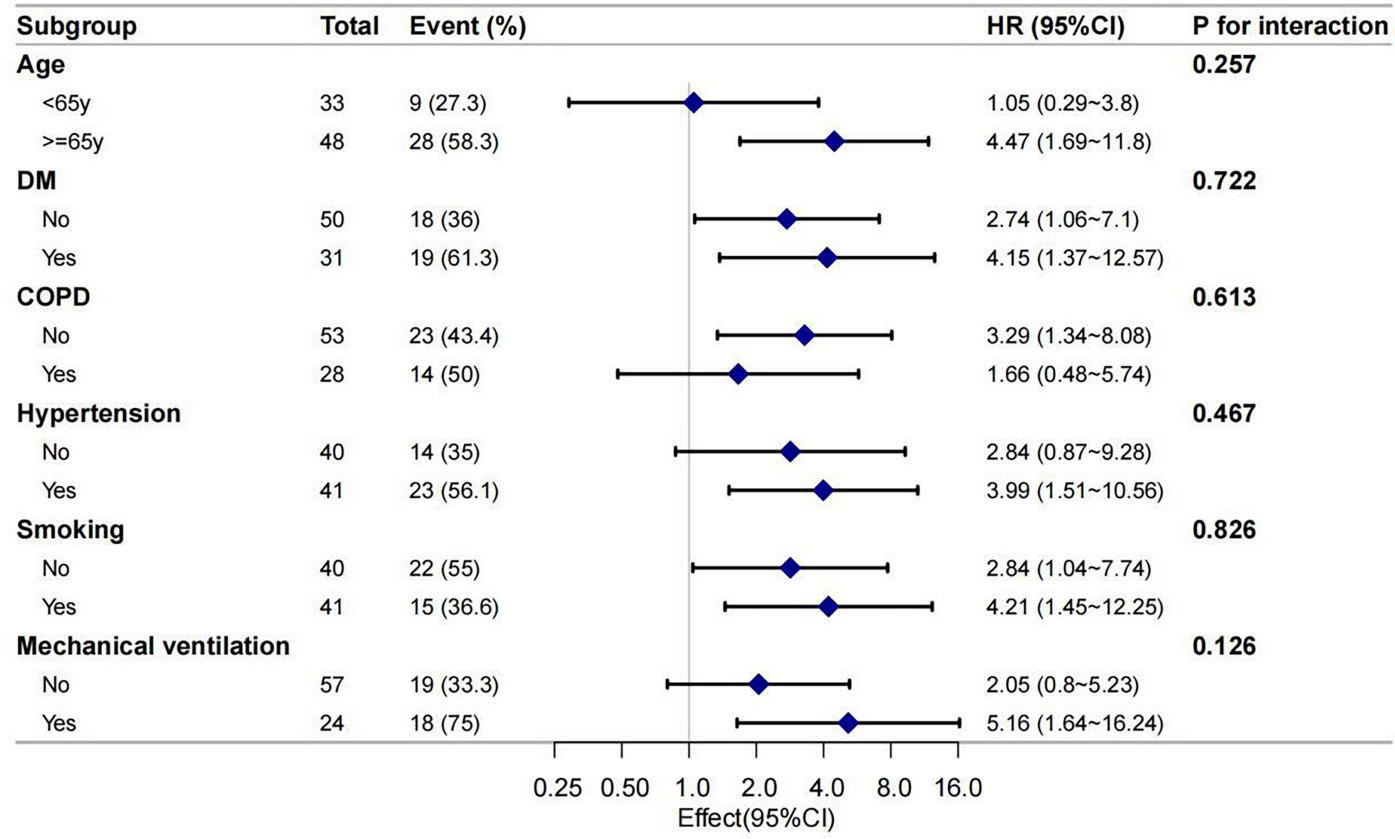
Forest plot for subgroup analyses of the association between concurrent pretreatment GM positivity and 30-day mortality. GM, galactomannan; COPD, chronic obstructive pulmonary disease; DM, diabetes mellitus.

Subgroup analyses were conducted to examine whether the association between concurrent pretreatment GM positivity and 30-day mortality differed across predefined strata (Figure 3). The stratified analyses included age (<65 vs. ≥65 years; P for interaction = 0.257), DM (P for interaction = 0.722), COPD (P for interaction = 0.613), hypertension (P for interaction = 0.467), smoking status (P for interaction = 0.826), and mechanical ventilation (P for interaction = 0.126). No significant interactions were observed across subgroups (all P for interaction > 0.05), indicating that the association between concurrent pretreatment GM positivity and 30-day mortality was generally consistent.

## 4 Discussion

In this retrospective cohort study of non-neutropenic IPA patients without underlying malignancy or solid organ transplantation, concurrent pretreatment positivity for both serum and BALF GM was independently associated with an increased risk of 30-day all-cause mortality. The mortality rate was markedly higher in patients with dual GM positivity than in those who were negative for both (45.7% vs. 13.6%; *P* < 0.001).

It is noteworthy that patients with concurrent GM positivity exhibited more severe laboratory abnormalities and higher disease severity scores at baseline. This finding is biologically plausible, as galactomannan reflects active fungal growth and may serve as a surrogate of invasive disease burden rather than a random biomarker (7, 17, 19, 20). Importantly, this baseline imbalance does not indicate selection bias, as GM testing was systematically performed prior to antifungal therapy in all enrolled patients, and initial antifungal regimens (voriconazole as first-line) were largely standardized. Instead, disease severity was explicitly addressed through multivariable adjustment, in which concurrent pretreatment GM positivity remained independently associated with 30-day mortality (adjusted hazard ratio, 3.07; 95% CI, 1.5–6.29; *P* = 0.002).

The clinical relevance of our findings is underscored by the growing recognition that IPA in non-neutropenic patients without malignancy or solid organ transplantation is frequently underdiagnosed and carries substantial mortality. This is often attributable to atypical clinical presentations, which can lead to delays in diagnosis and treatment (21–23). Serum and BALF GM testing serve as key microbiological criteria for IPA diagnosis, and GM levels are thought to reflect the fungal burden (7, 17). However, the prognostic significance of concurrent pretreatment positivity for both serum and BALF GM in this specific patient population has remained unclear (17, 18). To address this knowledge gap, we specifically evaluated the prognostic value of dual GM positivity—defined per 2020 EORTC/MSGERC (7) criteria (serum GM ODI ≥ 0.7 and BALF GM ODI ≥ 0.8)—while excluding patients with isolated positivity in either compartment (ODI ≥ 1.0). This study design, contrasting strictly defined dually positive and dually negative patients, was implemented to minimize population heterogeneity and thereby clarify the association. Our analysis consequently provides robust evidence that concurrent pretreatment GM positivity identifies an independent high-risk phenotype, which is associated with a significantly increased mortality risk (adjusted HR = 3.07).

Our findings should be interpreted against the backdrop of prior studies reporting inconsistent conclusions. For instance, Bartoletti et al. identified higher BALF GM levels as an independent predictor of mortality in COVID-19-associated pulmonary aspergillosis (CAPA) (24), whereas a large multicenter cohort found no such association for BALF GM positivity alone when culture results were negative (18). Similarly, studies by Hurt et al. (25), Ergün et al. (17) did not establish a clear prognostic role for BALF GM, while Fisher et al. reported that serum GM predicted mortality in hematopoietic stem cell transplant recipients (26). This heterogeneity likely stems from variations in study populations, host factors, and diagnostic criteria.

Interestingly, although the GM-positive group exhibited a higher positivity rate of IFA, no significant difference was observed in sputum culture results between the two groups. This discrepancy is not unexpected, as sputum culture is known to have limited sensitivity and poor specificity for invasive pulmonary aspergillosis, particularly in non-neutropenic patients (27, 28). In contrast, IFA detects fungal hyphae, while GM reflects antigens released during active fungal proliferation, which may better reflect invasive disease activity rather than mere airway colonization (27, 29). Importantly, concurrent pretreatment GM positivity remained independently associated with 30-day mortality, supporting its prognostic relevance beyond conventional microbiological culture results.

Given that most prior studies evaluated serum or BALF GM in isolation, we hypothesized that a combined assessment might provide more robust prognostic information. Our study contributes to this discourse by proposing that the combined assessment of serum and BALF GM may help reconcile these discrepancies. We hypothesize that positivity in BALF GM may indicate a high local fungal burden, whereas serum GM positivity suggests subsequent systemic dissemination (17). Consequently, dual positivity likely identifies a distinct patient subgroup with a more aggressive disease phenotype, which provides a plausible biological explanation for the significantly worse outcomes we observed.

The 30-day all-cause mortality in this cohort was 29.6%, a rate comparable to the previous report (30) but lower than others (23, 31). This variation may be attributed to improved recognition of non-classical risk factors and heightened awareness of IPA in the context of influenza and COVID-19, which have likely facilitated earlier diagnosis and contributed to declining mortality in more recent cohorts (17–19, 23, 31–33). Furthermore, the systematic application of combined GM testing in our study—where all enrolled patients underwent pretreatment serum and BALF GM testing—may have promoted the earlier identification of high-risk patients (e.g., those with dual positivity). This, in turn, could have enabled intensified monitoring or treatment optimization, potentially improving overall outcomes. However, it is important to note that this study was not designed to evaluate the direct impact of GM testing on clinical decision-making. Moreover, mortality in IPA remains strongly influenced by underlying comorbidities, host immune status, and the timeliness of antifungal therapy (31–34).

Several limitations of this study warrant consideration. First, its retrospective, single-center design may introduce selection bias and residual confounding, despite our use of prespecified exclusion criteria and multivariable adjustment. Second, by restricting our comparison to patients with dual GM positivity versus those with dual negativity, we were unable to evaluate the incremental prognostic value of concurrent positivity over isolated positivity in a single compartment. Third, although we excluded cases with known causes of false-positive GM results (e.g., certain antibiotics or albumin infusions), undetermined factors might have influenced GM levels in some instances.

Moreover, virus-associated IPA, such as influenza-associated pulmonary aspergillosis (IAPA) or COVID-19-associated pulmonary aspergillosis (CAPA), represents an important clinical subtype. However, data for each subgroup were extremely limited in this study and insufficient for statistical analysis. Therefore, the potential impact of virus-associated pulmonary aspergillosis on clinical outcomes could not be specifically evaluated in the present study.

In addition, therapeutic drug monitoring of voriconazole was not systematically performed during the study period. Consequently, potential variability in voriconazole exposure could not be assessed and may have influenced treatment outcomes.

Furthermore, PCR was not systematically performed during the study period, as this test had not yet been incorporated into routine clinical practice at our center. Consequently, PCR results were available only for a subset of patients and could not be included in the present analysis. We acknowledge this as a limitation of the study.

Finally, the moderate sample size limited the statistical precision of our subgroup analyses, and the findings may not be fully generalizable to other patient populations or settings that employ different diagnostic thresholds or assay platforms.

Future research should include prospective, multicenter studies to validate our findings. Such investigations should also determine the incremental prognostic value of combined GM testing over single-compartment testing and evaluate whether risk stratification based on dual GM positivity can guide clinical management decisions—such as the intensification of antifungal therapy or more frequent monitoring—and ultimately translate into improved patient outcomes.

## 5 Conclusion

In conclusion, this study establishes that concurrent pretreatment positivity for both serum and BALF GM is an independent prognostic marker for increased 30-day all-cause mortality in non-neutropenic IPA patients without classic immunocompromising conditions. This combined assessment serves as an effective tool for early risk stratification, identifying a patient subgroup that might benefit from more vigilant monitoring and intensified management strategies.

## Data Availability

The original contributions presented in this study are included in this article/Supplementary material, further inquiries can be directed to the corresponding author.

## Footnotes

1
https://www.R-project.org
