# Supplementary material for: Concurrent pretreatment serum and BALF galactomannan positivity as a prognostic indicator in non-neutropenic invasive pulmonary aspergillosis without malignancy or solid organ transplantation: a retrospective cohort study
Source: Front Med (Lausanne). 2026 May 13;13:1809671. doi: 10.3389/fmed.2026.1809671 (PMC13212345; doi:10.3389/fmed.2026.1809671)
Supplement: Supplementary file 1 [file Table_1.docx]

| **Supplementary Table 1**  **Univariate Cox regression analysis of predictors for 30-day mortality** | | |
| --- | --- | --- |
| **Variables** | **HR（95%CI）** | **P value** |
|  |  |  |
| Age (years) | 1.01 (0.99,1.04) | 0.199 |
| Sex, male, n (%) | 0.96 (0.49,1.88) | 0.901 |
| Hypertension, n (%) | 1.8 (1.01,3.2) | 0.047 |
| DM, n (%) | 1.92 (1.09,3.38) | 0.024 |
| COPD, n (%) | 1.32 (0.74,2.37) | 0.347 |
| Bacterial.pneumonia, n (%) | 1.77 (0.99,3.13) | 0.052 |
| Smoking, n (%) | 0.57 (0.32,1.01) | 0.053 |
| Mechanical.ventilation, n (%) | 2.51 (1.42,4.43) | 0.002 |
| OI (mmHg) | 0.9986 (0.9957,1.0016) | 0.373 |
| APACHE II score, n (%) | 1.1 (1.06,1.13) | < 0.001 |
| ALB (g/L) | 0.91 (0.86,0.96) | < 0.001 |
| SCr (μmoI/L) | 1.0033 (1.0017,1.0049) | < 0.001 |
| Glu (mmol/L) | 1.06 (1.03,1.09) | < 0.001 |
| LDH (U/L) | 1.0008 (1,1.0016) | 0.04 |
| WBC (×109/L) | 1.04 (1.01,1.08) | 0.023 |
| Neu# (×109/L) | 1.05 (1.01,1.09) | 0.007 |
| Lym# (×109/L) | 1.16 (0.78,1.73) | 0.462 |
| HGB (g/L) | 0.99 (0.98,0.99) | 0.003 |
| PLT (×109/L) | 0.9976 (0.9942,1.001) | 0.17 |
| EO (×109/L) | 0.13 (0,5) | 0.27 |
| IL-6 (pg/ml) | 1.0001 (1,1.0002) | 0.016 |
| PCT (ng/ml) | 1.0013 (0.9951,1.0076) | 0.675 |
| CRP (mg/L) | 1.0041 (1.0012,1.0071) | 0.006 |
| IFA, n (%) | 0.31 (0.12,0.8) | 0.014 |
| SC, n (%) | 1.06 (0.59,1.88) | 0.852 |
| GM, n (%) | 4.15 (2.12,8.15) | < 0.001 |

DM, diabetes mellitus; COPD, chronic obstructive pulmonary disease; OI, oxygenation index; APACHE II,Acute Physiology and Chronic Health Evaluation II; ALB, albumin; SCr, serum creatinine; Glu, glucose; LDH, lactate dehydrogenase; WBC, white blood cell count; Neu#, neutrophil count; Lym#, lymphocyte count; HGB, hemoglobin; PLT, platelet count; EO, eosinophil count; IL-6, interleukin-6; PCT, procalcitonin; CRP, C-reactive protein; IFA, immunofluorescence assay; SC, sputum culture.

**Supplementary Table 2**

****Collinearity diagnostics and covariate selection based on stability assessment between crude and fully adjusted models****

| Term1 | coeff1 | Change.percentage1 | Term2 | coeff2 | Change.percentage2 | VIF | colinearity | select | select.VIF |
| --- | --- | --- | --- | --- | --- | --- | --- | --- | --- |
| Crude | 1.42 | Ref. | Full | 0.97 | Ref. | 1.292 | 0 | Ref. | Ref. |
| ALB | 1.25 | -12 | ALB | 1.01 | 4.2 | 1.575 | 0 | Yes | Yes |
| SCr | 1.25 | -12.1 | SCr | 0.93 | -4.3 | 2.097 | 0 | Yes | Yes |
| Glu | 1.27 | -10.5 | Glu | 1.03 | 6.2 | 1.229 | 0 | Yes | Yes |
| HGB | 1.35 | -5.4 | HGB | 0.97 | -0.3 | 1.666 | 0 | No | No |
| Mechanical ventilation | 1.51 | 6.3 | Mechanical ventilation | 0.97 | -0.1 | 2.788 | 0 | No | No |
| Lym# | 1.42 | -0.6 | Lym# | 1.06 | 8.9 | 4.125 | 1 | No | Pending |
| CRP | 1.34 | -5.6 | CRP | 0.97 | -0.3 | 1.533 | 0 | No | No |
| Neu# | 1.34 | -6.2 | Neu# | 1.15 | 18.1 | 515.845 | 1 | Yes | Pending |
| DM | 1.41 | -0.8 | DM | 1 | 3.4 | 1.225 | 0 | No | No |
| Hypertension | 1.41 | -1.2 | Hypertension | 0.99 | 2.1 | 1.335 | 0 | No | No |
| WBC | 1.36 | -4.6 | WBC | 1.13 | 16.2 | 523.91 | 1 | Yes | Pending |
| IL-6 | 1.37 | -3.9 | IL-6 | 0.94 | -3.6 | 1.487 | 0 | No | No |
| APACHE II score | 1.34 | -5.6 | APACHE.IIscore | 0.92 | -5.2 | 2.864 | 0 | No | No |
| IFA | 1.29 | -9.4 | IFA | 1.02 | 5.3 | 1.187 | 0 | No | No |

ALB, albumin; SCr, serum creatinine; Glu, glucose; HGB, hemoglobin;Lym#, lymphocyte count; CRP, C-reactive protein;Neu#, neutrophil count;DM, diabetes mellitus; WBC, white blood cell count;IL-6, interleukin-6;APACHE II,Acute Physiology and Chronic Health Evaluation II;IFA, immunofluorescence assay;Ref.,Reference;VIF,Variance Inflation Factor.
